# Optical Fredkin gate assisted by quantum dot within optical cavity under vacuum noise and sideband leakage

**DOI:** 10.1038/s41598-020-61938-8

**Published:** 2020-03-20

**Authors:** Min-Sung Kang, Jino Heo, Seong-Gon Choi, Sung Moon, Sang-Wook Han

**Affiliations:** 1 Center for Quantum Information, Korea Institute of Science and Technology (KIST), Seoul, 02792 Republic of Korea; 2 Korean Intellectual Property Office, Government Complex Daejeon Building 4, 189, Cheongsa-ro, Seo-gu, Daejeon, 35208 Republic of Korea; 3 College of Electrical and Computer Engineering, Chungbuk National University, Chungdae-ro 1, Seowon-Gu, Cheongju, Republic of Korea; 4 Institute of Natural Science, Korea University, Sejong, 30091 Republic of Korea; 5 Division of Nano and Information Technology, Korea Institute of Science and Technology School, Korea University of Science and Technology, Seoul, 02792 Republic of Korea

**Keywords:** Quantum information, Quantum optics, Quantum optics, Quantum information, Quantum information

## Abstract

We propose a deterministic Fredkin gate which can accomplish controlled-swap operation between three-qubit states. The proposed Fredkin gate consists of a photonic system (single photon) and quantum dots (QDs) confined in single-sided cavities (two electron spin states). In our scheme, the control qubit is the polarization state of the single photon, and two electron spin states in QDs play the role of target qubits (swapped states by control qubit). The interaction between a photon and an electron of QD within the cavity (QD-cavity system) significantly affects the performance of Fredkin gate. Thus, through the analysis of the QD-cavity system under vacuum noise and sideband leakage, we demonstrate that reliable interaction and performance of the QD-cavity system with photonic state (photon) can be acquired in our scheme. Consequently, the Fredkin gate proposed in this paper can be experimentally implemented with high feasibility and efficiency.

## Introduction

Quantum controlled operations play critical roles in quantum information processing schemes, such as quantum computation^1–7^ and quantum communication^8–15^, to accomplish a reliable performance with high efficiency. One of the universal quantum operations is controlled-NOT (CNOT) gate, which can perform the spin-flipping of a target qubit with regard to the state of a control qubit, and has been researched theoretically and experimentally^16–22^. However, when constructing multi-qubit controlled operations (gates) with CNOT gates and single qubit gates, the error probability of controlled operation, or the realization difficulty, increases in multi-qubit controlled gates.

Many researchers have proposed to directly design or realize multi-qubit controlled gates, such as Fredkin gate^23–26^, Toffoli gate^27,28^, and universal multi-qubit gates^29–32^. The Fredkin gate, which is a controlled-swap gate, can be widely applicable in various quantum information processing schemes (quantum communication^33–36^ and quantum computation^37–40^).

To obtain scheme for quantum information processing with the reliable performance, the most important task is to maintain the coherence of quantum state when to operate the procedure of multi-qubit controlled operations. In quantum dot (QD)-cavity system, which consists of an injected excess electron and a confined negatively charged exciton (X^−^) in optical cavity^2,4,11–13,15,41–54^, quantum information can be well stored for a long electron-spin coherence time (T_2_^e^~μs)^43,45,46,55–57^ and a limited spin relaxation period (T_1_^e^~ms)^42,50,58,59^ against the influence (decoherence effect) of environment. Therefore, several diverse quantum information processing schemes have been designed between photons and electrons in the QD-cavity system, as quantum controlled gates^21,24–26,30,31,47–49,52,60^, quantum communications^10–13,15,42^, and quantum entanglement^2,4,44,53,54,61–63^.

In this paper, we represent an optical Fredkin gate, which can perform a controlled-swap operation, using QD-cavity system and linearly optical devices. Because our gate employs the interactions between a photon (control qubit) and two electrons (target qubits) inside the QD-cavity systems (QD within a single-sided optical cavity), the long coherence time of target qubits (swapped electron spin states) can be achieved from the storage of quantum information in QD, for the reliable performance of the Fredkin gate. Subsequently, for the reliable Fredkin gate, we demonstrate the high efficiency and reliable performance of the interaction of photon-electron in QD under the vacuum noise in the QD-dipole operation, and leaky modes (sideband leakage and absorption) in the cavity mode through our analysis^51,54,64–66^. Consequently, the proposed Fredkin gate using QD-cavity systems has the feasibility and the reliable performance to experimentally realize the controlled-swap gate under the vacuum noise and leaky modes (sideband leakage and absorption).

## Optical Fredkin Gate via Quantum dot within a Single-sided Optical Cavity

### Interaction of photon and QD-cavity system

**Figure 1:**
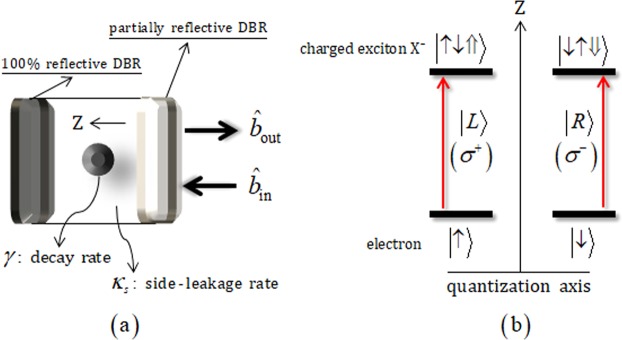
(**a**) A singly charged QD inside a single-sided cavity interacting with a photon (input and output field operators: ${\hat{b}}_{in}$ and ${\hat{b}}_{out}$). And the z axis means the quantization axis for angular momentum in cavity. (**b**) Interaction between a polarization of incident photon and a spin state of excess electron in QD. The photon $|L\rangle$ or *σ* ^+^ ($|R\rangle$ or *σ*^−^), which is propagated along the direction of the z axis, creates the transition to the charged exciton as |↑〉→|↑↓⇑〉 (|↓〉→|↑↓⇓〉) according to the spin selection rule, where |↑〉≡|+1/2〉, |↑〉≡|−1/2〉 are the spin states of the excess electron, and $|\Uparrow \rangle ,\,|\Downarrow \rangle \,({J}_{z}=+\,3/2,\,-\,3/2)$ represent heavy-hole spin states.

The interaction of a QD-cavity system consists of a singly charged QD confined in a single-sided optical cavity^2,4,11–13,15,41–54^. In Fig. 1(a), a QD-cavity system is schematically represented with two GaAs/Al(Ga)As distributed Bragg reflectors (DBRs). The bottom DBR is partially reflective and the top DBR is 100% reflective (single-sided cavity). ${\hat{b}}_{{\rm{i}}{\rm{n}}}$ and ${\hat{b}}_{{\rm{o}}{\rm{u}}{\rm{t}}}$ are the input and output field operators, respectively. QD is confined at the center of the single-sided cavity where *κ*_*s*_ (the side-leakage rate of the cavity mode) and *γ* (the decay rate of a negatively charged exciton, X^−^, which consists of two electrons bound to one hole. Figure 1(b) shows the interaction of QD-cavity system between an incident photon (described as ${\hat{b}}_{{\rm{i}}{\rm{n}}}$) and an excess electron injected into the QD, following the Pauli exclusion principle. If the spin state of the excess electron in the QD is in the state |↑〉(|↓〉), the polarization $|L\rangle \,(|R\rangle )$ of a photon can drive the state |↑↓⇑〉 (|↑↓⇓) of X^−^. In the approximation of weak excitation, after the interaction of the incident photon and QD-cavity system, we can obtain the reflection coefficient, *R*(*ω*), of the photon and the QD-cavity system from the Heisenberg equation of motion^67^, with the ground state in the QD (〈${\hat{\sigma }}_{z}$〉 = −1) for the steady state, due to the spin selection rule, as follows:

1 $$R(\omega )=\frac{[i({\omega }_{{{\rm{X}}}^{-}}-\omega )+\gamma /2][i({\omega }_{c}-\omega )-\kappa /2+{\kappa }_{s}/2]+{g}^{2}}{[i({\omega }_{{{\rm{X}}}^{-}}-\omega )+\gamma /2][i({\omega }_{c}-\omega )+\kappa /2+{\kappa }_{s}/2]+{g}^{2}},$$

where ${\omega }_{{{\rm{X}}}^{-}}$, *ω*_*c*_, and *ω* are the frequencies of X^−^, cavity mode, and external field, respectively. Additionally, *κ* and *g* are the decay rate of the cavity mode and the coupling strength between X^−^ and cavity mode, assuming the resonant interaction, *ω*_*c*_ = ${\omega }_{{{\rm{X}}}^{-}}$. When the spin state of the excess electron is in the state $|\,\uparrow \,\rangle \,(|\,\downarrow \,\rangle )$, the polarization of the photon $|L\rangle \,(|R\rangle )$ drive the hot cavity, according to the coupled QD with the cavity, while the photon having the polarization, $|R\rangle \,(|L\rangle )$, feels the cold cavity (the QD is uncoupled to the cavity). In this case, the reflection coefficients, *R*_h_ (hot cavity) and *R*_0_ (cold cavity), are given by:

2 $$\begin{array}{c}(g\ne 0)\to \,R(\omega )={R}_{{\rm{h}}}(\omega )\equiv |{r}_{{\rm{h}}}(\omega )|\exp [i{\varphi }_{{\rm{rh}}}(\omega )]=\frac{[i({\omega }_{c}-\omega )+\gamma /2][i({\omega }_{c}-\omega )-\kappa /2+{\kappa }_{s}/2]+{g}^{2}}{[i({\omega }_{c}-\omega )+\gamma /2][i({\omega }_{c}-\omega )+\kappa /2+{\kappa }_{s}/2]+{g}^{2}},\\ (g=0)\,\to \,{R}_{0}(\omega )\equiv |{r}_{0}(\omega )|\exp [i{\varphi }_{{\rm{r0}}}(\omega )]=\frac{i({\omega }_{c}-\omega )-\kappa /2+{\kappa }_{s}/2}{i({\omega }_{c}-\omega )+\kappa /2+{\kappa }_{s}/2},\end{array}$$

where |*r*_h_| (|*r*_0_|) and ${\varphi }_{{\rm{rh}}}\equiv \text{arg}[{R}_{{\rm{h}}}]\,({\varphi }_{{\rm{r0}}}\equiv \text{arg}[{R}_{0}])$ are the reflectance and the phase shift of the hot (cold) cavity. Thus, the reflection operator $\hat{{\rm{R}}}({\rm{\omega }})$ is expressed as:

3 $$\hat{{\rm{R}}}(\omega )=|{r}_{{\rm{h}}}(\omega )|{e}^{i{\varphi }_{{\rm{rh}}}(\omega )}(|R\rangle \langle R|\,\otimes \,|\,\downarrow \,\rangle \langle \,\downarrow \,|+|L\rangle \langle L|\,\otimes \,|\,\uparrow \,\rangle \langle \,\uparrow \,|)+|{r}_{0}(\omega )|{e}^{i{\varphi }_{{\rm{r0}}}(\omega )}(|R\rangle \langle R|\,\otimes \,|\,\uparrow \,\rangle \,\langle \,\uparrow \,|+|L\rangle \langle L|\,\otimes \,|\,\downarrow \,\rangle \langle \,\downarrow \,|).$$

In the QD-cavity system, when we take the experimental parameters as a small side-leakage rate, *κ*_*s*_ (*κ*_*s*_ << *κ*), and the strong coupling strength *g* >> (*κ*, *γ*) with small *γ* (~ several μ eV)^68–71^, the reflection operator can be given as:

4 $$\omega -{\omega }_{c}=0\,\to \,\hat{{\rm{R}}}\approx |R\rangle \langle R|\,\otimes \,|\,\downarrow \,\rangle \langle \,\downarrow \,|\,+\,|L\rangle \langle L|\,\otimes \,\,|\,\uparrow \,\rangle \langle \,\uparrow \,|\,-\,|R\rangle \langle R|\,\otimes \,|\,\uparrow \,\rangle \langle \,\uparrow \,|\,-\,|L\rangle \langle L|\,\otimes \,|\,\downarrow \,\rangle \langle \,\downarrow \,|,$$

where the reflectances and phase shifts are |*r*_h_(*ω*)| = |*r*_0_(*ω*)|≈1 and *φ*_rh_(*ω*)≈0, *φ*_r0_(*ω*)≈*π* for *g*/*κ* = 2.4, and *γ*/*κ* = 0.1 with *κ*_*s*_ → 0 and *ω* = *ω*_*c*_ (frequencies: external field = cavity mode)^2,4,11–13,15,41–54^. According to the reflection operator, Eq. 4, the result of the interaction between the photon and the QD-cavity is expressed as:

5 $$|R\rangle |\,\uparrow \,\rangle \mathop{\Rightarrow }\limits^{{\rm{QD}}}-|R\rangle |\,\uparrow \,\rangle ,|R\rangle |\,\downarrow \,\rangle \mathop{\Rightarrow }\limits^{{\rm{QD}}}|R\rangle |\,\downarrow \,\rangle ,|L\rangle |\,\uparrow \,\rangle \mathop{\Rightarrow }\limits^{{\rm{QD}}}|L\rangle |\,\uparrow \,\rangle ,|L\rangle |\,\downarrow \,\rangle \mathop{\Rightarrow }\limits^{{\rm{QD}}}-|L\rangle |\,\downarrow \,\rangle .$$

### Theoretical circuit of Fredkin gate

**Figure 2:**
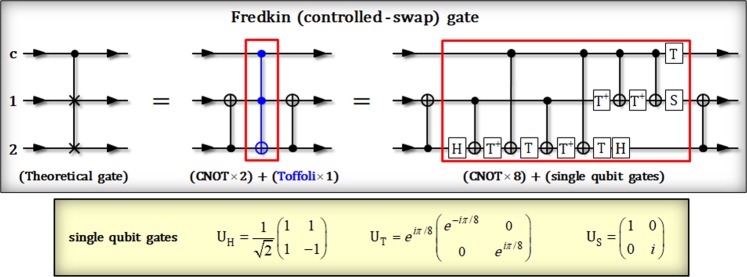
This plot describes a theoretical circuit of Fredkin gate on the end of left side. And a circuit of the middle consists of two CNOT gates (two-qubit gate)^16–22^ and a Toffoli gate (three-qubit gate)^27,28^. Also, in last circuit (on the end of right side), the Toffoli gate can be decomposed by six CNOT gates and single qubit gates (H, T, and S), as described in the red box. These circuits are equivalent to perform the operation (controlled-swap) of Fredkin gate.

First, we introduce the theoretical Fredkin (controlled-swap operation) gate^23–26^. In Fig. 2, the Fredkin (theoretical) gate can perform a controlled-swap operation between three qubits of a control qubit, c, and two target qubits, 1 and 2 (which are swapped by the state of control qubit). The operation of Fredkin gate is given by:

6 $$|{{\boldsymbol{\Psi }}}_{{\rm{in}}}\rangle =(\alpha {|0\rangle }_{{\rm{c}}}+\beta {|1\rangle }_{{\rm{c}}})\otimes {|\varphi \rangle }_{1}{|\psi \rangle }_{2}\,\mathop{\to }\limits^{{\rm{Fredkin}}\,{\rm{gate}}}\,|{{\boldsymbol{\Psi }}}_{{\rm{out}}}\rangle =\alpha {|0\rangle }_{{\rm{c}}}\otimes {|\varphi \rangle }_{1}{|\psi \rangle }_{2}\,+\,\beta {|1\rangle }_{{\rm{c}}}\otimes {|\psi \rangle }_{1}{|\varphi \rangle }_{2},$$

where $\alpha {|0\rangle }_{{\rm{c}}}+\beta {|1\rangle }_{{\rm{c}}}$ is the control qubit with *|α*|^2^ + *|β*|^2^ = 1. Additionally, ${|\varphi \rangle }_{1}$ and ${|\psi \rangle }_{2}$ are arbitrary quantum states (target qubits). This result means that if the state of control qubit is in the ${|1\rangle }_{{\rm{c}}}$, two target states 1 and 2 are exchanged for each other (controlled-swap). In Fig. 2, the Fredkin gate (theoretical circuit) can be implemented the multi-qubit (two or three) controlled gates and the single qubit gates for the experimental realization. If we attempt to design Fredkin gate using CNOTs and Toffoli gates, we can reduce the number of controlled operations needed (the middle circuit in Fig. 2). However, it is challengeable to directly implement Toffoli gate (three-qubit controlled gate) in practice. Thus, in this point of view, the methods of using only two-qubit controlled gates for the Fredkin gate have the experimental advantage, despite the number of controlled gates (or operations) increase (the end of right side in Fig. 2). For example, in Fig. 2, though the middle circuit has three controlled operations, the task to realize Toffoli gate is experimentally difficult. Meanwhile, in the circuit of right side, the number of controlled operations needed is eight interactions. And also this circuit can guarantee the feasibility for the implementation, due to consisting of the only two-qubit controlled gates. Therefore, we will propose the method of utilizing two-qubit (a photon and an electron in QD: QD-cavity system) controlled gates to design an optical Fredkin gate with the experimental feasibility and reducing the number of controlled operations needed.

### Optical Fredkin gate using QD-cavity systems

**Figure 3:**
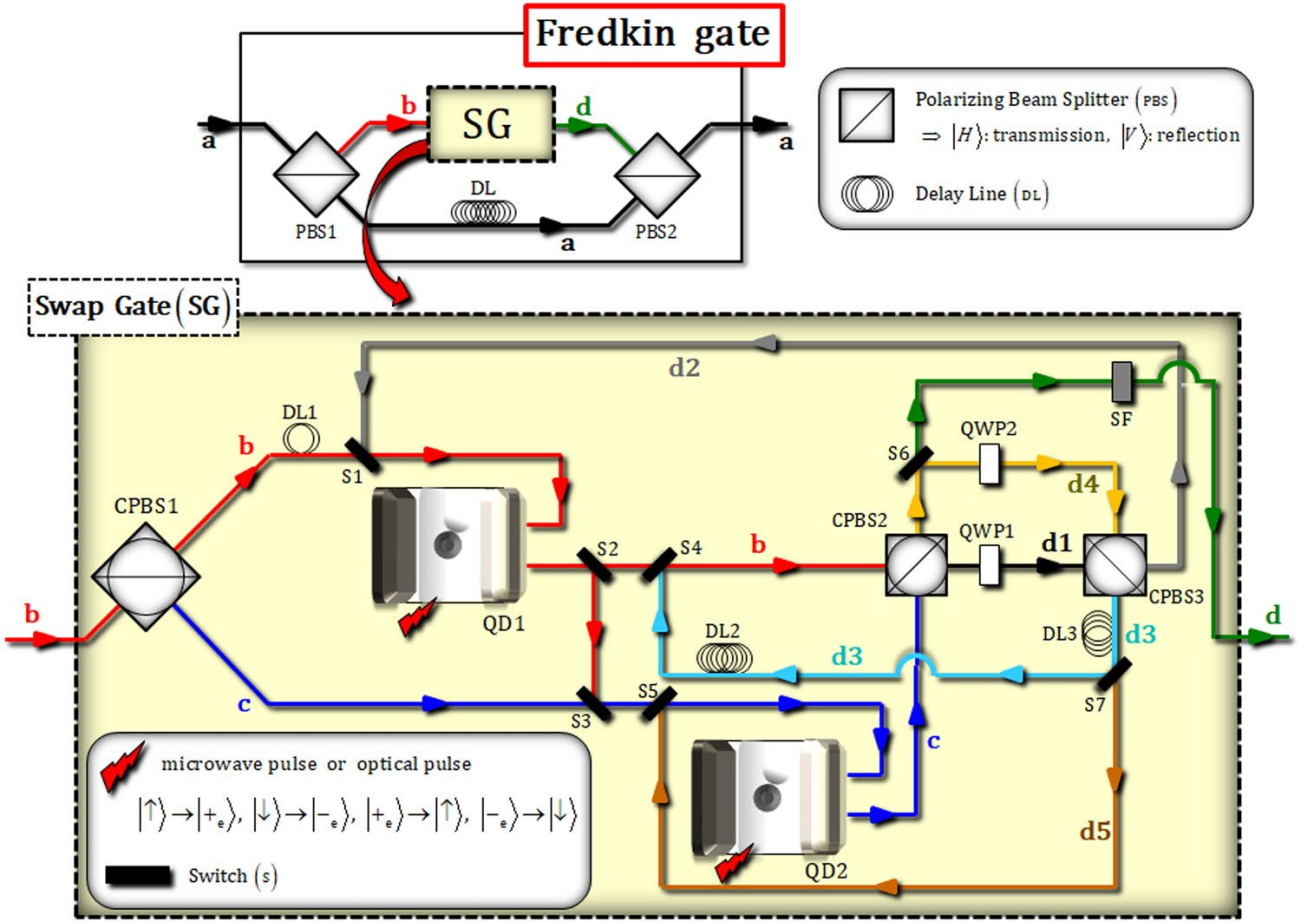
Schematic of an optical Fredkin gate: For a controlled-swap operation, the swap gate (SG) having two QD-cavity systems is a critical component (light-yellow box). A single photon P and two electrons (1 and 2) in QD play the roles of control qubit and target qubits, respectively. A Hadamard operation is operated on the electron spin state by using a microwave pulse or an optical pulse^13,76,77^. DL is a delay line, which can conduct the time-delay of a photon for the synchronization, and switches perform to alter (reflect or transfer) the path of photon according to time table.

Figure 3 shows an optical scheme, the Fredkin gate, to perform the controlled-swap operation as Eq. 6. The proposed Fredkin gate consists of a swap gate (SG), which has two QD-cavity systems (QD 1 and 2), and linearly optical devices, as described in Fig. 3. Let us assume the initial (input) state, ${|{{\boldsymbol{\Psi }}}_{{\rm{in}}}\rangle }_{{\rm{P}}12}$, of a Fredkin gate, as follows:

7 $${|{{\boldsymbol{\Psi }}}_{{\rm{in}}}\rangle }_{{\rm{P}}12}=(\alpha {|H\rangle }_{{\rm{P}}}^{{\rm{a}}}+\beta {|V\rangle }_{{\rm{P}}}^{{\rm{a}}})\otimes ({\alpha }_{1}{|\uparrow \rangle }_{1}+{\beta }_{1}{|\downarrow \rangle }_{1})({\alpha }_{2}{|\uparrow \rangle }_{2}+{\beta }_{2}{|\downarrow \rangle }_{2}),$$

where |*α*_i_|^2^ + |*β*_i_|^2^ = 1. The control qubit is the flying photon P, and two target qubits are two electron spin states 1 and 2, which are stationary states confined within the QD-cavity systems (QD 1 and 2), respectively. We also define the relations between the polarizations of photon and electron spin states, as:

8 $$({\rm{polarization}}):|H\rangle \equiv (|R\rangle +|L\rangle )/\sqrt{2}\,,\,|V\rangle \equiv (|R\rangle -|L\rangle )/\sqrt{2}\,,\,({\rm{spin}}\,{\rm{state}}):\,|{\pm }_{{\rm{e}}}\,\rangle \equiv (|\,\uparrow \,\rangle \pm |\,\downarrow \,\rangle )/\sqrt{2},$$

where the linear polarization, $\{|H\rangle ,\,|V\rangle \}$, and the circular polarization, $\{|R\rangle ,\,|L\rangle \}$ of photon. As described in Fig. 3, after the state, ${|{{\boldsymbol{\Psi }}}_{{\rm{in}}}\rangle }_{{\rm{P}}12}$, passes through PBS1, the state will be changed to:

9 $$\begin{array}{c}{|{{\boldsymbol{\Psi }}}_{{\rm{in}}}\rangle }_{{\rm{P}}12}\,\mathop{\to }\limits^{{\rm{PBS1}}}\,{|{{\boldsymbol{\Psi }}}_{1}\rangle }_{{\rm{P}}12}=\alpha {|H\rangle }_{{\rm{P}}}^{{\rm{a}}}\otimes ({\alpha }_{1}{|\uparrow \rangle }_{1}+{\beta }_{1}{|\downarrow \rangle }_{1})({\alpha }_{2}{|\uparrow \rangle }_{2}+{\beta }_{2}{|\downarrow \rangle }_{2})\\ \,+\,\beta {|V\rangle }_{{\rm{P}}}^{{\rm{b}}}\otimes ({\alpha }_{1}{|\uparrow \rangle }_{1}+{\beta }_{1}{|\downarrow \rangle }_{1})({\alpha }_{2}{|\uparrow \rangle }_{2}+{\beta }_{2}{|\downarrow \rangle }_{2}),\end{array}$$

where the path of the photon P is split into two paths, a and b. Subsequently, the photon state on the path b of the state ${|{{\boldsymbol{\Psi }}}_{1}\rangle }_{{\rm{P}}12}$ is applied to SG, while the state on path a is delayed by DL, in Fig. 3. In SG, the state on path b will have three interactions (three times) with the QD-cavity systems (QD 1 and 2).

### [First cycle: time Table (1) {t = 0~t_1_}]

**Figure 4:**
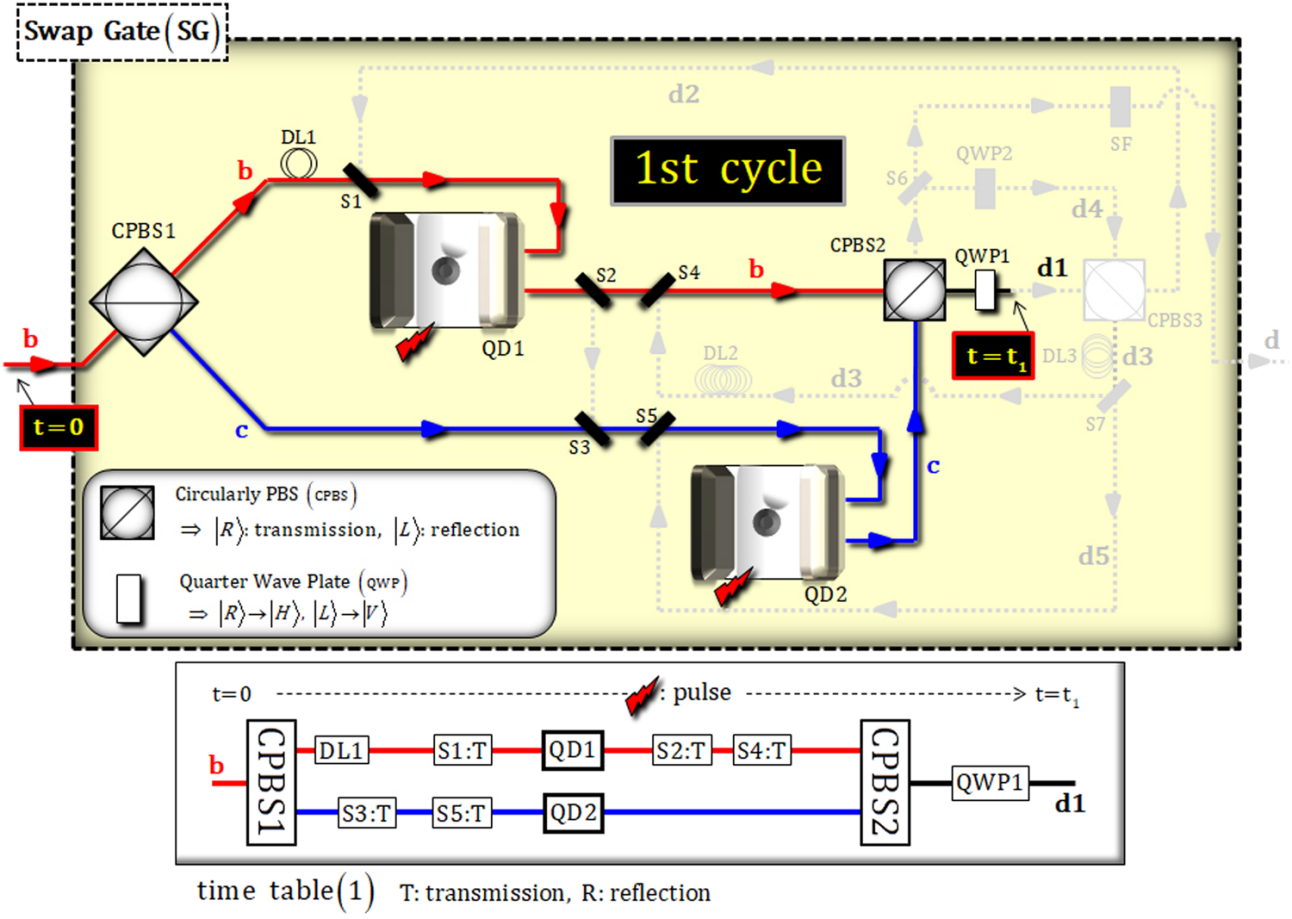
First cycle in SG: The photon state, which is split into two paths (**b,c**) by CBPS1, interacts with two QD-cavity systems (QD 1 and 2) for the first cycle. As described in time Table (1), the procedure (DL, switches, and pulse) is conducted for time (0~t_1_).

Figure 4 represents the interaction between the photon state and two electron spin states (1 and 2) in the first cycle. DL1 and the switches (1, 2, 3, 4, and 5) are operated according to the time Table (1)), for the interval from t = 0 to t_1_. After the interaction of two QD-cavity systems (QD 1 and 2) with the photon, the state, ${|{{\boldsymbol{\Psi }}}_{1}\rangle }_{{\rm{P}}12}$, is transformed to:

10 $$\begin{array}{c}{|{{\boldsymbol{\Psi }}}_{1}\rangle }_{{\rm{P}}12}\mathop{\longrightarrow }\limits^{{\rm{CPBS1}}\,:\,({\rm{DL1}},\,{\rm{S1}})\,\& \,({\rm{S3}},\,{\rm{S5}})\,:\,{\rm{QD1}}\,\& \,{\rm{QD2}}}\\ \begin{array}{cc}\to \,{|{{\boldsymbol{\Psi }}}_{2}\rangle }_{{\rm{P}}12}= & \alpha {|H\rangle }_{{\rm{P}}}^{{\rm{a}}}\otimes ({\alpha }_{1}{|\uparrow \rangle }_{1}+{\beta }_{1}{|\downarrow \rangle }_{1})({\alpha }_{2}{|\uparrow \rangle }_{2}+{\beta }_{2}{|\downarrow \rangle }_{2})\\  & +\,\frac{\beta }{\sqrt{2}}[{|R\rangle }_{{\rm{P}}}^{{\rm{b}}}\otimes (-{\alpha }_{1}{|\uparrow \rangle }_{1}+{\beta }_{1}{|\downarrow \rangle }_{1})({\alpha }_{2}{|\uparrow \rangle }_{2}+{\beta }_{2}{|\downarrow \rangle }_{2})\\  & +{|L\rangle }_{{\rm{P}}}^{{\rm{c}}}\otimes ({\alpha }_{1}{|\uparrow \rangle }_{1}+{\beta }_{1}{|\downarrow \rangle }_{1})({\alpha }_{2}{|\uparrow \rangle }_{2}-{\beta }_{2}{|\downarrow \rangle }_{2})],\end{array}\end{array}$$

where the interaction between the photon and the QD-cavity system is described from the reflection operator, $\hat{{\rm{R}}}$, in Eqs. 4 and 5. Afterward, the pulses (microwave pulse or optical pulse in Fig. 3) are applied to the two electrons, 1 and 2, of QD1 and QD2 in the state ${|{{\boldsymbol{\Psi }}}_{2}\rangle }_{{\rm{P}}12}$. Subsequently, we can obtain the output state, ${|{{\boldsymbol{\Psi }}}_{3}\rangle }_{{\rm{P}}12}$ (t = t_1_), of the first cycle in SG after the state ${|{{\boldsymbol{\Psi }}}_{2}\rangle }_{{\rm{P}}12}$ passes through CPBS2 and QWP1, as follows:

11 $$\begin{array}{c}{|{{\boldsymbol{\Psi }}}_{2}\rangle }_{{\rm{P}}12}\,\mathop{\longrightarrow }\limits^{({\rm{pulse}}):\,({\rm{S2}},{\rm{S4}}):\,{\rm{CPBS2}}:\,{\rm{QWP1}}}\\ \begin{array}{cc}\to {|{{\boldsymbol{\Psi }}}_{3}\rangle }_{{\rm{P}}12}= & \alpha {|H\rangle }_{{\rm{P}}}^{{\rm{a}}}\otimes ({\alpha }_{1}{|{+}_{{\rm{e}}}\rangle }_{1}+{\beta }_{1}{|{-}_{{\rm{e}}}\rangle }_{1})({\alpha }_{2}{|{+}_{{\rm{e}}}\rangle }_{2}+{\beta }_{2}{|{-}_{{\rm{e}}}\rangle }_{2})\\  & +\,\frac{\beta }{\sqrt{2}}[{|H\rangle }_{{\rm{P}}}^{{\rm{d1}}}\otimes (-{\alpha }_{1}{|{+}_{{\rm{e}}}\rangle }_{1}+{\beta }_{1}{|{-}_{{\rm{e}}}\rangle }_{1})({\alpha }_{2}{|{+}_{{\rm{e}}}\rangle }_{2}+{\beta }_{2}{|{-}_{{\rm{e}}}\rangle }_{2})\\  & +\,{|V\rangle }_{{\rm{P}}}^{{\rm{d1}}}\otimes ({\alpha }_{1}{|{+}_{{\rm{e}}}\rangle }_{1}+{\beta }_{1}{|{-}_{{\rm{e}}}\rangle }_{1})({\alpha }_{2}{|{+}_{{\rm{e}}}\rangle }_{2}-{\beta }_{2}{|{-}_{{\rm{e}}}\rangle }_{2})].\end{array}\end{array}$$

### [Second cycle: time table (2) {t = t_1_~t_2_}]

**Figure 5:**
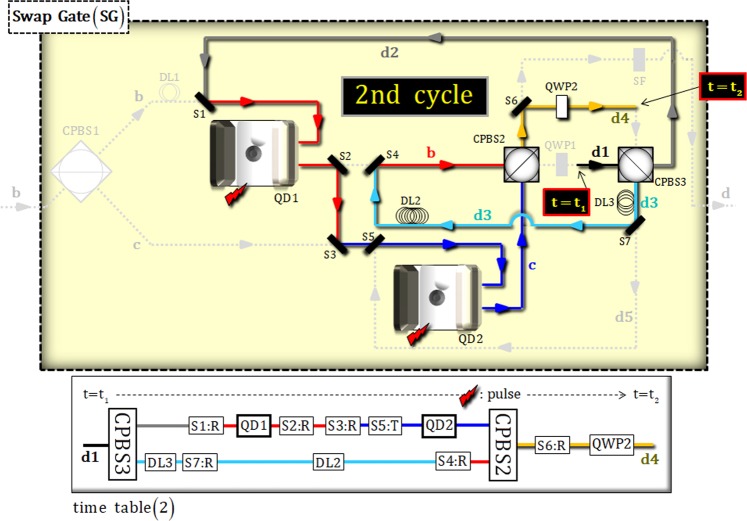
Second cycle in SG: After CPBS3 (t_1_), the state on path d2 (excepting path d3) sequentially interacts with two QD-cavity systems (QD 1 and 2) for the second cycle. The procedure (DLs, switches, and pulse) is operated, due to time table (2), for time (t_1_~t_2_).

Figure 5 represents the interaction between the photon state on path d2 and two electron spin states (1 and 2) in the second cycle. The DLs and switches (1, 2, 3, 4, 5, 6, and 7) are operated according to the time table (2), for the interval from t = t_1_ to t_2_. After the state, ${|{{\boldsymbol{\Psi }}}_{3}\rangle }_{{\rm{P}}12}$, passes through CPBS3, the state will be changed to:

12 $$\begin{array}{c}{|{{\boldsymbol{\Psi }}}_{3}\rangle }_{{\rm{P}}12}\,\mathop{\longrightarrow }\limits^{{\rm{CPBS3}}}\\ \begin{array}{cc}\to \,{|{{\boldsymbol{\Psi }}}_{4}\rangle }_{{\rm{P}}12}= & \alpha {|H\rangle }_{{\rm{P}}}^{{\rm{a}}}\otimes ({\alpha }_{1}{|{+}_{{\rm{e}}}\rangle }_{1}+{\beta }_{1}{|{-}_{{\rm{e}}}\rangle }_{1})({\alpha }_{2}{|{+}_{{\rm{e}}}\rangle }_{2}+{\beta }_{2}{|{-}_{{\rm{e}}}\rangle }_{2})\\  & +\,\beta [{|R\rangle }_{{\rm{P}}}^{{\rm{d2}}}\otimes (-{\alpha }_{1}{\beta }_{2}{|{+}_{{\rm{e}}}\rangle }_{1}{|{-}_{{\rm{e}}}\rangle }_{2}+{\beta }_{1}{\alpha }_{2}{|{-}_{{\rm{e}}}\rangle }_{1}{|{+}_{{\rm{e}}}\rangle }_{2})\\  & +{|L\rangle }_{{\rm{P}}}^{{\rm{d3}}}\otimes (-{\alpha }_{1}{\alpha }_{2}{|{+}_{{\rm{e}}}\rangle }_{1}{|{+}_{{\rm{e}}}\rangle }_{2}+{\beta }_{1}{\beta }_{2}{|{-}_{{\rm{e}}}\rangle }_{1}{|{-}_{{\rm{e}}}\rangle }_{2})].\end{array}\end{array}$$

Afterward, the interactions, due to the reflection operator $\hat{{\rm{R}}}$ (Eqs. 4 and 5), of two QD-cavity systems (QD 1 and 2) are sequentially performed to the state, ${|{{\boldsymbol{\Psi }}}_{4}\rangle }_{{\rm{P}}12}$, as follows:

13 $$\begin{array}{c}{|{{\boldsymbol{\Psi }}}_{4}\rangle }_{{\rm{P}}12}\,\mathop{\longrightarrow }\limits^{{\rm{S1}}\,\& \,({\rm{DL3}},\,{\rm{S7}})\,:\,{\rm{QD1}}:\,({\rm{S2}},\,{\rm{S3}},\,{\rm{S5}})\,\& \,{\rm{DL2}}\,:\,{\rm{QD2}}\,\& \,{\rm{S4}}}\\ \begin{array}{cc}\to \,{|{{\boldsymbol{\Psi }}}_{5}\rangle }_{{\rm{P}}12}= & \alpha {|H\rangle }_{{\rm{P}}}^{{\rm{a}}}\otimes ({\alpha }_{1}{|{+}_{{\rm{e}}}\rangle }_{1}+{\beta }_{1}{|{-}_{{\rm{e}}}\rangle }_{1})({\alpha }_{2}{|{+}_{{\rm{e}}}\rangle }_{2}+{\beta }_{2}{|{-}_{{\rm{e}}}\rangle }_{2})\\  & +\,\beta [{|R\rangle }_{{\rm{P}}}^{{\rm{c}}}\otimes (-{\alpha }_{1}{\beta }_{2}{|{-}_{{\rm{e}}}\rangle }_{1}{|{+}_{{\rm{e}}}\rangle }_{2}+{\beta }_{1}{\alpha }_{2}{|{+}_{{\rm{e}}}\rangle }_{1}{|{-}_{{\rm{e}}}\rangle }_{2})\\  & +{|L\rangle }_{{\rm{P}}}^{{\rm{b}}}\otimes (-{\alpha }_{1}{\alpha }_{2}{|{+}_{{\rm{e}}}\rangle }_{1}{|{+}_{{\rm{e}}}\rangle }_{2}+{\beta }_{1}{\beta }_{2}{|{-}_{{\rm{e}}}\rangle }_{1}{|{-}_{{\rm{e}}}\rangle }_{2})].\end{array}\end{array}$$

where two electron spin states, which are linked with the photon state on path c, are exchanged (swapped), compared with the state ${|{{\boldsymbol{\Psi }}}_{4}\rangle }_{{\rm{P}}12}$ in Eq. 12. Then, the pulses (microwave pulse or optical pulse in Fig. 3) are applied to the two electrons, 1 and 2, of QD1 and QD2 in the state ${|{{\boldsymbol{\Psi }}}_{5}\rangle }_{{\rm{P}}12}$. After the state, ${|{{\boldsymbol{\Psi }}}_{5}\rangle }_{{\rm{P}}12}$, passes through CPBS2 and QWP2, we can obtain the output state, ${|{{\boldsymbol{\Psi }}}_{6}\rangle }_{{\rm{P}}12}$ (t = t_2_), of the second cycle in SG, as follows:

14 $$\begin{array}{c}{|{{\boldsymbol{\Psi }}}_{5}\rangle }_{{\rm{P}}12}\,\mathop{\longrightarrow }\limits^{({\rm{pulse}})\,:\,{\rm{CPBS2}}\,:\,{\rm{S6}}\,:\,{\rm{QWP2}}}\\ \to {|{{\boldsymbol{\Psi }}}_{6}\rangle }_{{\rm{P}}12}=\alpha {|H\rangle }_{{\rm{P}}}^{{\rm{a}}}\otimes ({\alpha }_{1}{|\uparrow \rangle }_{1}+{\beta }_{1}{|\downarrow \rangle }_{1})({\alpha }_{2}{|\uparrow \rangle }_{2}+{\beta }_{2}{|\downarrow \rangle }_{2})\\ \,+\,\beta [{|H\rangle }_{{\rm{P}}}^{{\rm{d4}}}\otimes (\,-{\alpha }_{1}{\beta }_{2}{|\downarrow \rangle }_{1}{|\uparrow \rangle }_{2}+{\beta }_{1}{\alpha }_{2}{|\uparrow \rangle }_{1}{|\downarrow \rangle }_{2})+{|V\rangle }_{{\rm{P}}}^{{\rm{d4}}}\otimes (\,-{\alpha }_{1}{\alpha }_{2}{|\uparrow \rangle }_{1}{|\uparrow \rangle }_{2}+{\beta }_{1}{\beta }_{2}{|\downarrow \rangle }_{1}{|\downarrow \rangle }_{2})].\end{array}$$

### [Third cycle: time table (3) {t = t_2_~t_3_}]

**Figure 6:**
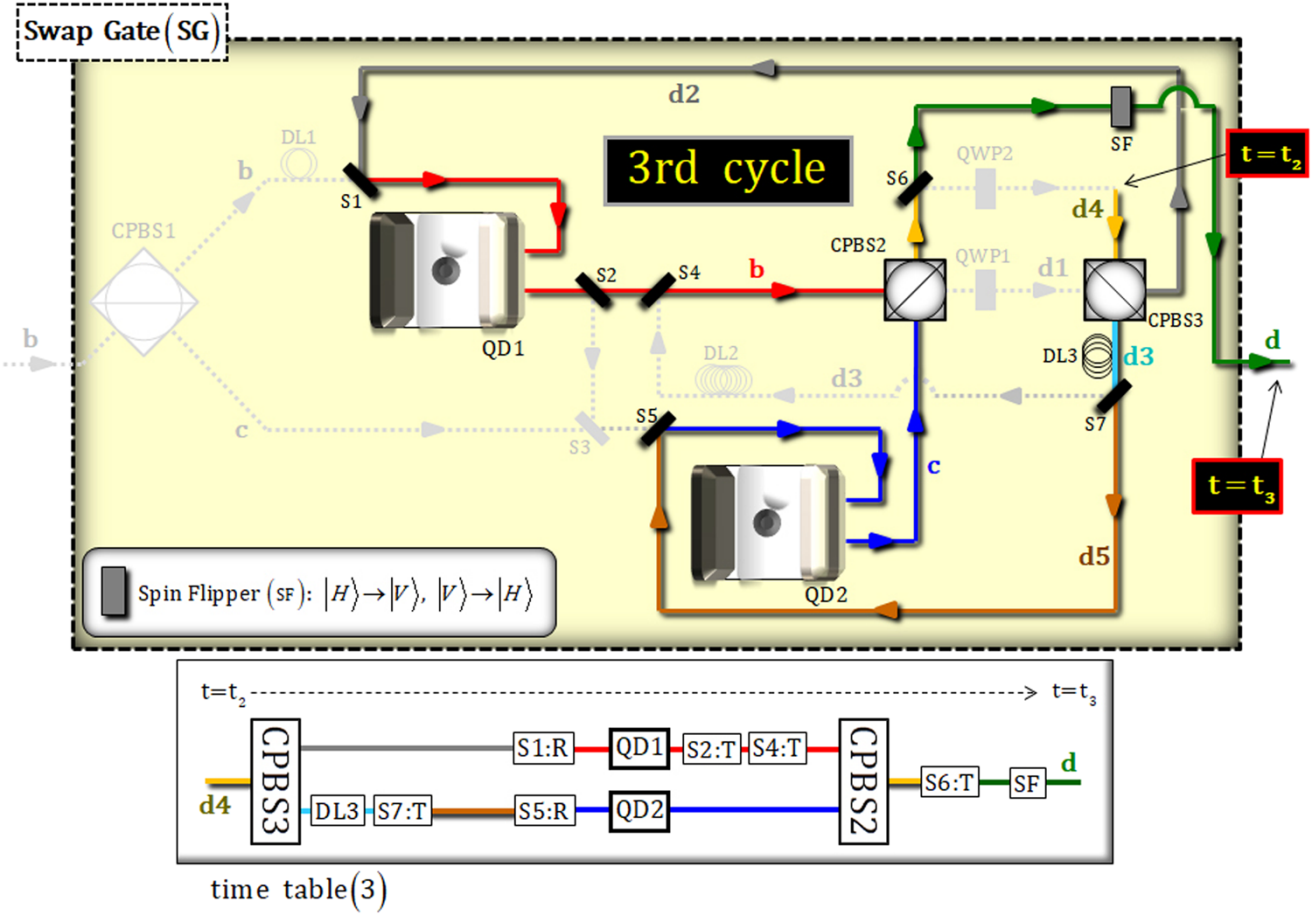
Third cycle in SG: After CPBS3 (t_2_), the photon state interacts with two QD-cavity systems (QD 1 and 2) for the third cycle. The procedure (DL and switches) is operated, as described in time table (3), for time (t_2_~t_3_).

In Fig. 6, the interactions between the photon and two QD-cavity systems (QD 1 and 2) are operated in the third (final) cycle for time (t_2_~t_3_). The procedure of DL and switches (1, 2, 4, 5, 6, and 7) is performed in accordance with the time table (3). The output state, ${|{{\boldsymbol{\Psi }}}_{6}\rangle }_{{\rm{P}}12}$, from the second cycle is transformed after passing through CPBS3, as follows:

15 $$\begin{array}{c}{|{{\boldsymbol{\Psi }}}_{6}\rangle }_{{\rm{P}}12}\,\mathop{\longrightarrow }\limits^{{\rm{CPBS3}}}\\ \begin{array}{cc}\to \,{|{{\boldsymbol{\Psi }}}_{7}\rangle }_{{\rm{P}}12}= & \alpha {|H\rangle }_{{\rm{P}}}^{{\rm{a}}}\otimes ({\alpha }_{1}{|\uparrow \rangle }_{1}+{\beta }_{1}{|\downarrow \rangle }_{1})({\alpha }_{2}{|\uparrow \rangle }_{2}+{\beta }_{2}{|\downarrow \rangle }_{2})\\  & -\,\frac{\beta }{\sqrt{2}}[{|R\rangle }_{{\rm{P}}}^{{\rm{d3}}}\otimes ({\alpha }_{2}{|\uparrow \rangle }_{1}+{\beta }_{2}{|\downarrow \rangle }_{1})({\alpha }_{1}{|\uparrow \rangle }_{2}-{\beta }_{1}{|\downarrow \rangle }_{2})\\  & -{|L\rangle }_{{\rm{P}}}^{{\rm{d2}}}\otimes ({\alpha }_{2}{|\uparrow \rangle }_{1}-{\beta }_{2}{|\downarrow \rangle }_{1})({\alpha }_{1}{|\uparrow \rangle }_{2}+{\beta }_{1}{|\downarrow \rangle }_{2})].\end{array}\end{array}$$

Then, the photon of the state ${|{{\boldsymbol{\Psi }}}_{7}\rangle }_{{\rm{P}}12}$ interacts with two QD-cavity systems (QD 1 and 2), according to Eqs. 4 and 5, as follows:

16 $$\begin{array}{c}{|{{\boldsymbol{\Psi }}}_{7}\rangle }_{{\rm{P}}12}\mathop{\longrightarrow }\limits^{{\rm{S1}}\,\& \,({\rm{DL3}},\,{\rm{S7}},\,{\rm{S5}})\,:\,{\rm{QD1}}\,\& \,{\rm{QD2}}\,:\,({\rm{S2}},\,{\rm{S4}})}\\ \begin{array}{cc}\to \,{|{{\boldsymbol{\Psi }}}_{8}\rangle }_{{\rm{P}}12}= & \alpha {|H\rangle }_{{\rm{P}}}^{{\rm{a}}}\otimes ({\alpha }_{1}{|\uparrow \rangle }_{1}+{\beta }_{1}{|\downarrow \rangle }_{1})({\alpha }_{2}{|\uparrow \rangle }_{2}+{\beta }_{2}{|\downarrow \rangle }_{2})\\  & +\frac{\beta }{\sqrt{2}}({|R\rangle }_{{\rm{P}}}^{{\rm{c}}}+{|L\rangle }_{{\rm{P}}}^{{\rm{b}}})\otimes ({\alpha }_{2}{|\uparrow \rangle }_{1}+{\beta }_{2}{|\downarrow \rangle }_{1})({\alpha }_{1}{|\uparrow \rangle }_{2}+{\beta }_{1}{|\downarrow \rangle }_{2}).\end{array}\end{array}$$

Finally, after the state ${|{{\boldsymbol{\Psi }}}_{8}\rangle }_{{\rm{P}}12}$ passes through CPBS2, S6, and SF, we can obtain the output state, ${|{{\boldsymbol{\Psi }}}_{9}\rangle }_{{\rm{P}}12}$ (*t* = *t*_3_), of the third (final) cycle in SG, as follows:

17 $$\begin{array}{c}{|{{\boldsymbol{\Psi }}}_{8}\rangle }_{{\rm{P}}12}\mathop{\longrightarrow }\limits^{{\rm{CPBS2}}\,:\,{\rm{S6}}\,:\,{\rm{SF}}}\\ \begin{array}{cc}\to {|{{\boldsymbol{\Psi }}}_{9}\rangle }_{{\rm{P}}12}= & \alpha {|H\rangle }_{{\rm{P}}}^{{\rm{a}}}\otimes ({\alpha }_{1}{|\uparrow \rangle }_{1}+{\beta }_{1}{|\downarrow \rangle }_{1})({\alpha }_{2}{|\uparrow \rangle }_{2}+{\beta }_{2}{|\downarrow \rangle }_{2})\\  & +\beta {|V\rangle }_{{\rm{P}}}^{{\rm{d}}}\otimes ({\alpha }_{2}{|\uparrow \rangle }_{1}+{\beta }_{2}{|\downarrow \rangle }_{1})({\alpha }_{1}{|\uparrow \rangle }_{2}+{\beta }_{1}{|\downarrow \rangle }_{2}).\end{array}\end{array}$$

Here, the state ${|{{\boldsymbol{\Psi }}}_{9}\rangle }_{{\rm{P}}12}$ from SG (through the cycles, three times) shows that two electron spin states in QD 1 and QD 2 are swapped with each other, according to the polarization of photon P (${|V\rangle }_{{\rm{P}}}$ is to swap two electron spin states). Consequently, after the state, ${|{{\boldsymbol{\Psi }}}_{9}\rangle }_{{\rm{P}}12}$, passes PBS2 in Fig. 3, we can show that our optical Fredkin gate in Fig. 3 can generate the operation of controlled-swap through the final state, ${|{{\boldsymbol{\Psi }}}_{{\rm{out}}}\rangle }_{{\rm{P}}12}$, as follows:18

Compared with the input state, ${|{{\boldsymbol{\Psi }}}_{{\rm{in}}}\rangle }_{{\rm{P}}12}$ in Eq. 7, this result, ${|{{\boldsymbol{\Psi }}}_{{\rm{out}}}\rangle }_{{\rm{P}}12}$ (output state), means that the controlled-swap operation, which performs the exchange of two states of target qubits (electron spin states 1 and 2) in the case of ${|V\rangle }_{{\rm{P}}}$ (control qubit: photon’s polarization), is conducted by the proposed Fredkin gate by utilizing the QD-cavity systems.

So far, we have proposed an optical Fredkin gate, consisted of the QD-cavity systems, to feasibly exploit the controlled-swap gate for the high efficiency and the reliable performance. In our scheme, two electrons 1 and 2 confined within QD-cavity systems (QD1 and QD2) play the roles of the target qubits. When a flying photon P (control qubit) interacts with the electrons respectively, the result of this interaction is identical with a controlled-phase operation (two-qubit controlled gate), due to the reflection operator $\hat{{\rm{R}}}(\omega )$ in Eq. 3. Thus, from each cycle of the three cycles in Figs. 4–6, we can calculate the total number of two-qubit controlled operations, as six interactions (a cycle has two interactions, as described in Figs. 4–6). Compared with the Fredkin gate having eight CNOT (two-qubit controlled) gates in Fig. 2, the proposed scheme in Fig. 3 can reduce the number of two-qubit controlled operations needed for the feasibility. Furthermore, in our Fredkin gate, swap gate (SG) having two QD-cavity systems (two target qubits) plays the role of main part to store quantum information with coherence and to optically interact the photon with the electron in QD. Thus, for the practical usage of Fredkin gate, the quantification for the interaction of the QD-cavity system (QD within a single-sided cavity) should be required by the analysis about the affections of the vacuum noise, sideband leakage, and absorption.

## Analysis of the Interaction of QD within Single-sided Cavity under Noise

**Figure 7:**
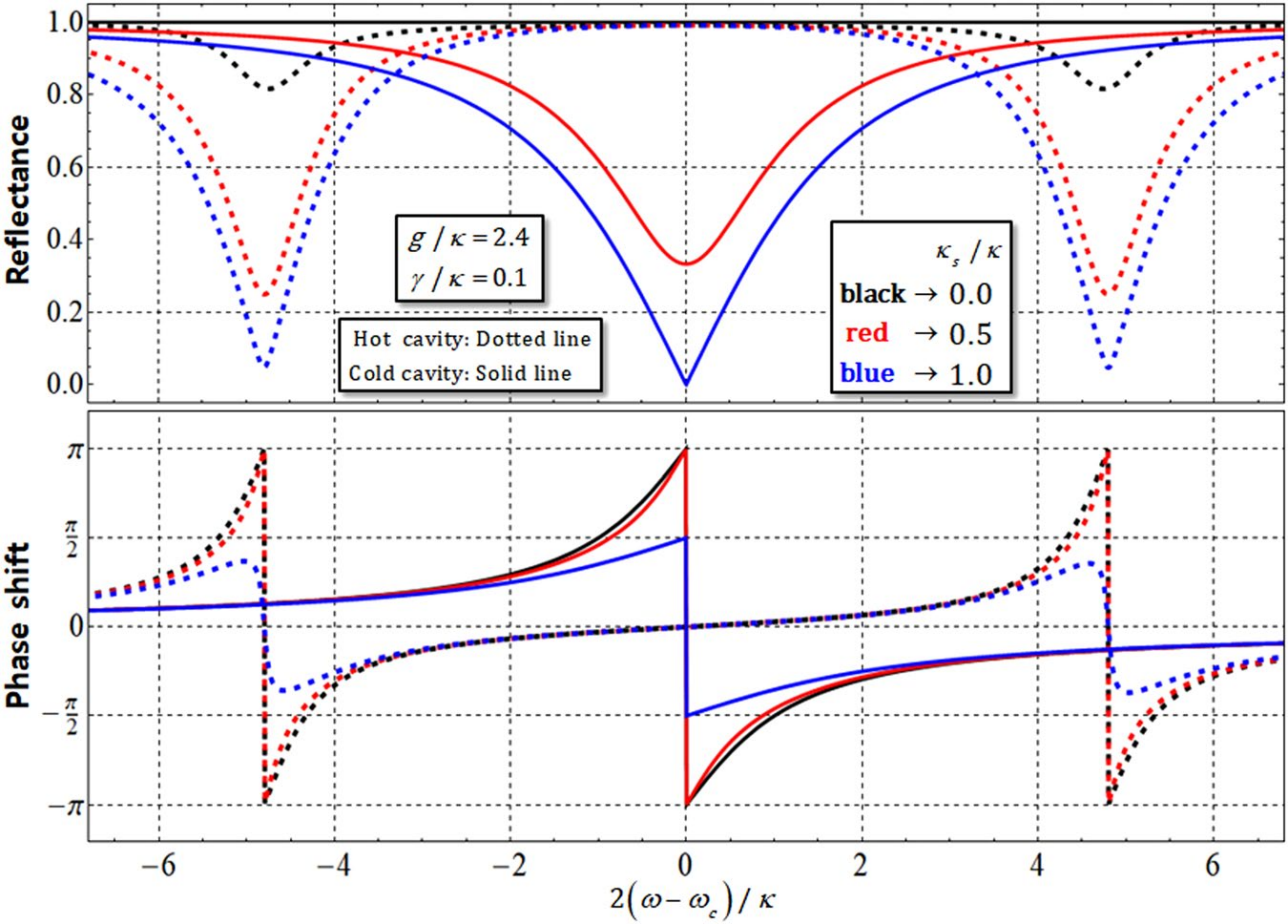
Graph of reflectances and phase shifts (|*r*_h_| and *φ*_rh_: hot cavity-dotted line), and (|*r*_0_| and *φ*_r0_: cold cavity-solid line) for difference in side-leakage rates, *κ*_*s*_/*κ* = 0.0, 0.5, and 1.0, with *g*/*κ* = 2.4, *γ*/*κ* = 0.1, and *ω*_*c*_ = *ω*. When *κ*_*s*_ is small (*κ*_*s*_ << *κ*) with strong coupling strength, *g* >> (*κ*, *γ*), the values of reflectances, |*r*_h_| and |*r*_0_|, approach 1, and also the value of phase shift, *φ*_rh_ (*φ*_r0_), is close to 0 (*π*) in the QD-cavity system.

In an optical Fredkin gate performing controlled-swap operation, the significant element is the QD-cavity system, which can interact with a flying photon and an electron confined within QD, inducing a difference in reflectances (|*r*_h_|, |*r*_0_|) and phase shifts (*φ*_rh_, *φ*_r0_) from Eqs. 2–4, according to the hot (coupled to) cavity and cold (uncoupled to) cavity. Figure 7 shows the reflectances and phase shifts of a reflected photon, which feels hot (*g* ≠ 0) and cold (*g* = 0) cavity, for frequency detuning, 2(*ω* − *ω*_*c*_)/*κ*, and the different side-leakage rates (*κ*_*s*_/*κ* = 0.0, 0.5, and 1.0) with fixed values of coupling strength, *g*/*κ* = 2.4, and decay rate, *γ*/*κ* = 0.1, of X^−^, from Eq. 2. When *κ*_*s*_ is negligible and *ω* = *ω*_*c*_ (the frequency, *ω*, of the external field, photon, can be adjusted to the identical frequency, *ω*_*c*_, of cavity mode), the values of reflectance and phase shift can be acquired, as |*r*_h_(*ω*)| = |*r*_0_(*ω*)| ≈ 1, *φ*_rh_(ω) = 0, and *φ*_r0_(*ω*) = *π*, as described in Fig. 7. The reflection operator, $\hat{{\rm{R}}}$, which is utilized in our Fredkin gate, can be given as Eq. 4, referring to the interaction, Eq. 5, of QD-cavity system.

In practice, for the high efficiency and reliable performance of the QD-cavity system, which can induce the interaction an input photon and an confined electron inside cavity, the affections of the vacuum noise, *N*(*ω*), of the QD-dipole and leaky modes, *S*(*ω*) (sideband leakage and absorption) should be quantified^51,54,64–66^. For the reflection coefficient, $\hat{{\rm{R}}}(\omega )$, with the noise, *N*(*ω*), and leakage, *S*(*ω*), coefficients, a solution of the Heisenberg equation of motion for a cavity field operator, *â*, a dipole operator, *σ̂*^−^, of X^−^, and the input-output relations^67^, is given by:

19 $$\begin{array}{c}\frac{d\hat{{a}}}{dt}\,=-\,\left[i({\omega }_{c}-\omega )+\frac{\kappa }{2}+\frac{{\kappa }_{s}}{2}\right]\hat{{a}}-g{\hat{\sigma }}_{-}-\sqrt{\kappa }{\hat{{b}}}_{{\rm{in}}}-\sqrt{{\kappa }_{s}}{\hat{S}}_{{\rm{in}}},\\ \frac{d{\hat{\sigma }}_{-}}{dt}\,=-\,\left[i({\omega }_{{{\rm{X}}}^{-}}-\omega )+\frac{\gamma }{2}\right]{\hat{\sigma }}_{-}-g{\hat{\sigma }}_{Z}\hat{{a}}+\sqrt{\gamma }{\hat{\sigma }}_{Z}\hat{N},\\ {\hat{{b}}}_{{\rm{out}}}\,={\hat{{b}}}_{{\rm{in}}}+\sqrt{\kappa }\hat{{a}},\,{\hat{S}}_{{\rm{out}}}={\hat{S}}_{{\rm{in}}}+\sqrt{\kappa }\hat{{a}},\end{array}$$

where ${\hat{S}}_{in}$ (${\hat{S}}_{out}$) is an input (output) field operator from leaky modes, due to sideband leakage and absorption in the cavity mode, and $\hat{N}$ is the vacuum noise operator for *σ̂*^−^. The output field operator, ${\hat{b}}_{out}$, of reflected photon, will be given by ${\hat{b}}_{{\rm{o}}{\rm{u}}{\rm{t}}}$ = *R*(*ω*)${\hat{b}}_{{\rm{i}}{\rm{n}}}$ + *S*(*ω*)${\hat{S}}_{{\rm{i}}{\rm{n}}}$ +* N*(*ω*)$\hat{S}$ from Eq. 19 in the weak excitation approximation^50,53^, with ω_*c*_ = ${\omega }_{{{\rm{X}}}^{-}}$ and 〈*σ̂*_*Z*_〉≈−1 (ground state in QD). The reflection, *R*(*ω*), noise, *N*(*ω*), and leakage, *S*(*ω*), coefficients can be expressed, according to a hot and cold cavity, as

20 $$\begin{array}{rcl}(g\ne 0) & \to  & N(\omega )={N}_{{\rm{h}}}(\omega )\equiv |{n}_{{\rm{h}}}(\omega )|\exp [i{\varphi }_{{\rm{nh}}}(\omega )]=\frac{\sqrt{\gamma \kappa }g}{[i({\omega }_{c}-\omega )+\gamma /2][i({\omega }_{c}-\omega )+\kappa /2+{\kappa }_{s}/2]+{g}^{2}},\\  & \to  & S(\omega )={S}_{{\rm{h}}}(\omega )\equiv |{s}_{{\rm{h}}}(\omega )|\exp [i{\varphi }_{{\rm{sh}}}(\omega )]=\frac{-\sqrt{{\kappa }_{s}\kappa }[i({\omega }_{c}-\omega )+\gamma /2]}{[i({\omega }_{c}-\omega )+\gamma /2][i({\omega }_{c}-\omega )+\kappa /2+{\kappa }_{s}/2]+{g}^{2}},\\ (g=0) & \to  & {N}_{0}(\omega )\equiv |{n}_{0}(\omega )|\exp [i{\varphi }_{{\rm{n0}}}(\omega )]=0,\\  & \to  & {S}_{0}(\omega )\equiv |{s}_{0}(\omega )|\exp [i{\varphi }_{{\rm{s0}}}(\omega )]=\frac{-\sqrt{{\kappa }_{s}\kappa }}{i({\omega }_{c}-\omega )+\kappa /2+{\kappa }_{s}/2},\end{array}$$

where the reflectances (hot: *R*_h_ and cold: *R*_0_) of *R*(*ω*), are written in Eq. 2. |*n*_h_| (|*n*_0_|), and *φ*_nh_ (*φ*_n0_) are noise rate and phase shift, corresponding vacuum noise the of QD-dipole, in hot (cold) cavity. Additionally, |*s*_h_| (|*s*_0_|) and *φ*_sh_ (*φ*_s0_) are leakage rate and phase shift, respectively, due to the sideband leakage and absorption for cavity mode, in a hot (cold) cavity. Thus, we can revise the reflection operator, $\hat{{\rm{R}}}(\omega )$ in Eq. 4, to practical reflection operator, $\hat{{\rm{R}}}$_P_(*ω*), including noise, *N*(*ω*), and leakage, *S*(*ω*), coefficients, as follows:

21 $$\begin{array}{rll}{\hat{{\rm{R}}}}_{{\rm{P}}}(\omega ) & = & [|{r}_{{\rm{h}}}(\omega )|{e}^{i{\varphi }_{{\rm{rh}}}(\omega )}+|{n}_{{\rm{h}}}(\omega )|{e}^{i{\varphi }_{{\rm{nh}}}(\omega )}+|{s}_{{\rm{h}}}(\omega )|{e}^{i{\varphi }_{{\rm{sh}}}(\omega )}](|R\rangle \langle R|\,\otimes \,|\,\downarrow \,\rangle \langle \,\downarrow \,|+|L\rangle \langle L|\,\otimes \,|\,\uparrow \,\rangle \langle \,\uparrow \,|)\\  & + & [|{r}_{0}(\omega )|{e}^{i{\varphi }_{{\rm{r0}}}(\omega )}+|{s}_{0}(\omega )|{e}^{i{\varphi }_{{\rm{s0}}}(\omega )}](|R\rangle \langle R|\,\otimes \,|\,\uparrow \,\rangle \langle \,\uparrow \,|+|L\rangle \langle L|\,\otimes \,|\,\downarrow \,\rangle \langle \,\downarrow \,|),\end{array}$$

where *R*_h_(*ω*) and *R*_0_(*ω*) are in Eq. 2, and ${N}_{0}(\omega )\equiv |{n}_{0}(\omega )|\exp [i{\varphi }_{{\rm{n0}}}(\omega )]=0$ is from Eq. 20.

**Figure 8:**
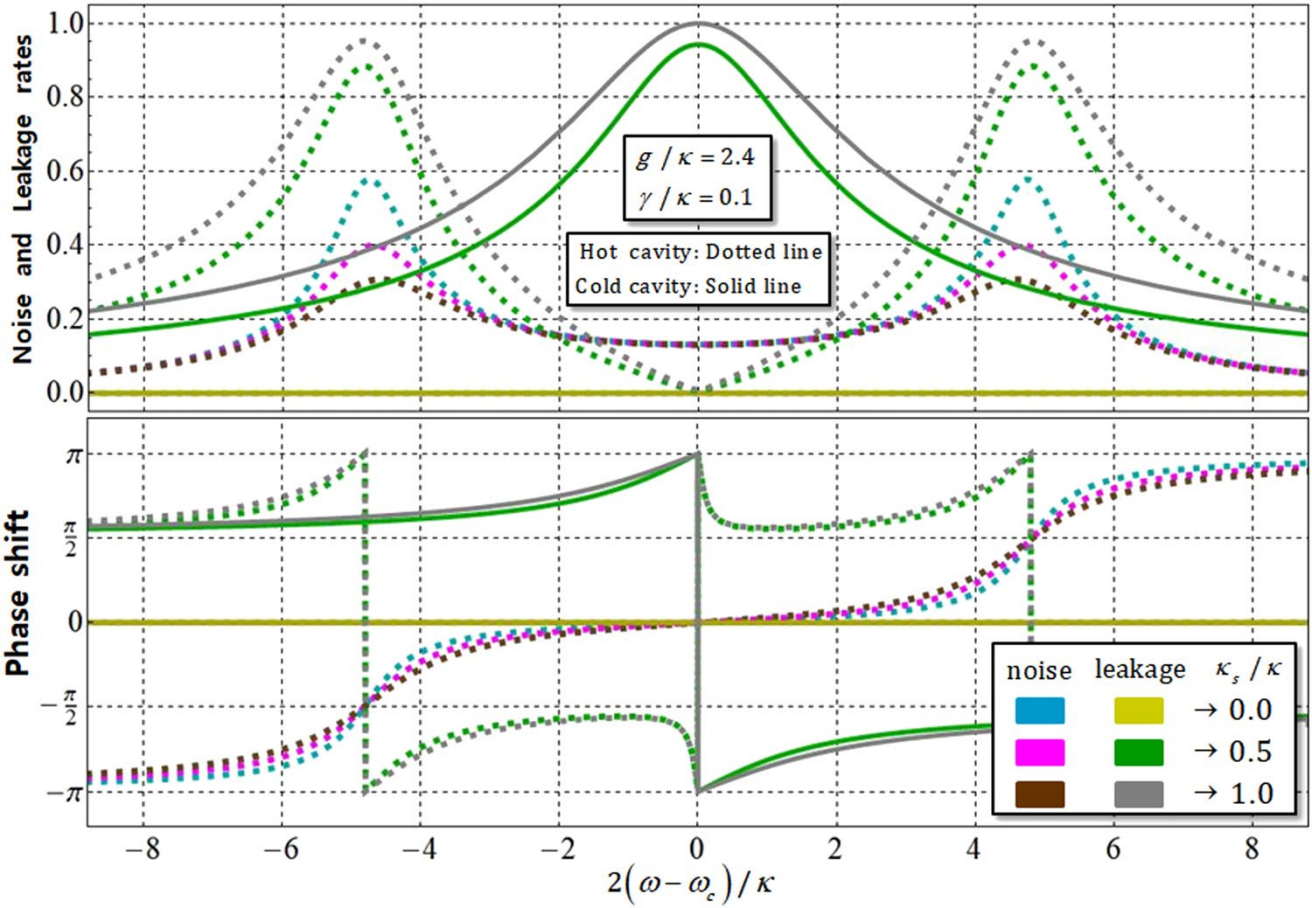
Graph of noise |*n*_h_| (|*n*_0_|), leakage |*s*_h_| (|*s*_0_|) rates, and phase shifts, *φ*_nh_ (*φ*_n0_) and *φ*_sh_ (*φ*_s0_), of hot (cold) cavity for difference in side-leakage rates, *κ*_*s*_/*κ* = 0.0, 0.5, and 1.0, with *g*/*κ* = 2.4, *γ*/*κ* = 0.1, and *ω*_*c*_ = *ω*. All rates and phase shifts (noise: |*n*_h_|, |*n*_0_|, *φ*_nh_, *φ*_n0_ and leakage: |*s*_h_|, |*s*_0_|, *φ*_sh_, *φ*_s0_) are negligible in QD-cavity system when *κ*_*s*_ is small (*κ*_*s*_ << *κ*), with strong coupling strength, *g* >> (*κ*, *γ*).

Figure 8 shows a graph of noise rate, phase shift (blue, magenta, and brown), and leakage rate, phase shift (yellow, green, and gray), from Eq. 20, for frequency detuning, 2(*ω*−*ω*_*c*_)/*κ*, and the different side-leakage rates (*κ*_*s*_/*κ* = 0.0, 0.5, and 1.0), when experimental parameters are strength coupling, *g*/*κ* = 2.4, and decay rate, *γ*/*κ* = 0.1, of X^−^ with *ω*_*c*_ = ${\omega }_{{{\rm{X}}}^{-}}$. When *κ*_*s*_ is negligible (*κ*_*s*_ → 0) and 2(*ω* − *ω*_*c*_)/*κ* = 0, we can obtain the values of noise and leakage rates, phase shifts as |*n*_h_| → 0, |*n*_0_| = |*s*_h_| = |*s*_0_| = 0, and *φ*_nh_ → 0, *φ*_n0_ = *φ*_sh_ = *φ*_s0_ = 0 in QD-cavity system, as described in Fig. 8. This result means that we can ignore the effects of the vacuum noise, *N*(*ω*), of QD-dipole operation and leakage (sideband leakage and absorption), *S*(*ω*), for high efficiency and reliable performance of QD-cavity system by choosing the parameters *g*/*κ* = 2.4 (strong coupling strength) and *κ*_*s*_≈0 (small side-leakage rate) with *γ*/*κ* = 0.1.

Therefore, we can analyze the efficiency and performance of a QD-cavity system to calculate the value of the average of fidelity (AoF) between two output states from the reflection operators $\hat{{\rm{R}}}(\omega )$ (Eq. 4: ideal case) and ${\hat{{\rm{R}}}}_{{\rm{P}}}(\omega )$ (Eq. 21: practical case). For example, let us assume an arbitrary input state (photon-electron) as $(\cos \,\theta |R\rangle +\,\sin \,\theta |L\rangle )\otimes (\cos \,\vartheta |\,\uparrow \,\rangle +\,\sin \,\vartheta |\,\downarrow \,\rangle )$ where cos^2^*θ* + sin^2^*θ* = cos^2^*ϑ* + sin^2^*ϑ* = 1.

After the interaction of QD-cavity system, we can show two kinds ($|{\phi }_{{\rm{Id}}}\rangle$: ideal case, and $|{\phi }_{{\rm{\Pr }}}\rangle$: practical case) of output states from the reflection operators ($\hat{{\rm{R}}}$ and ${\hat{{\rm{R}}}}_{{\rm{p}}}$) in Eqs. 4 and 21, as follows:

22 $$\begin{array}{ccc}|{\phi }_{{\rm{Id}}}\rangle  & = & (\cos \,\theta \,\sin \,\vartheta |R\rangle |\,\downarrow \,\rangle +\,\sin \,\theta \,\cos \,\vartheta |L\rangle |\,\uparrow \,\rangle )-(\cos \,\theta \,\cos \,\vartheta |R\rangle |\,\uparrow \,\rangle +\,\sin \,\theta \,\sin \,\vartheta |L\rangle |\,\downarrow \,\rangle ),\\ |{\phi }_{{\rm{pr}}}\rangle  & = & \frac{1}{\sqrt{{\rm{N}}}}[({R}_{{\rm{h}}}+{N}_{{\rm{h}}}+{S}_{{\rm{h}}})(\cos \,\theta \,\sin \,\vartheta |R\rangle |\,\downarrow \,\rangle +\,\sin \,\theta \,\cos \,\vartheta |L\rangle |\,\uparrow \,\rangle )\\  &  & +\,({R}_{0}+{S}_{0})(\cos \,\theta \,\cos \,\vartheta |R\rangle |\,\uparrow \,\rangle +\,\sin \,\theta \,\sin \,\vartheta |L\rangle |\,\downarrow \,\rangle )],\end{array}$$

where N = |*R*_h_ + *N*_h_ + *S*_h_|^2^(cos ^2^*θ* sin ^2^*ϑ* + sin ^2^*θ* cos ^2^*ϑ*) + |*R*_0_ + *S*_0_|^2^(cos^2^*θ* cos^2^*ϑ* + sin^2^*θ* sin^2^*ϑ*). Then, the AoF between two output states ($|{\phi }_{{\rm{Id}}}\rangle$ and $|{\phi }_{{\rm{\Pr }}}\rangle$) of QD-cavity system is given by:

23 $${\rm{AoF}}\equiv \frac{1}{4{\pi }^{2}}{\int }_{0}^{2\pi }{\int }_{0}^{2\pi }|\sqrt{\langle {\phi }_{{\rm{Id}}}|{\phi }_{{\rm{\Pr }}}\rangle \langle {\phi }_{{\rm{\Pr }}}|{\phi }_{{\rm{Id}}}\rangle }|d\theta d\vartheta .$$

**Figure 9:**
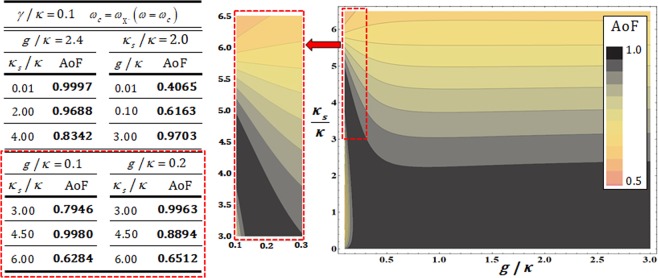
The left table shows the values of AoF for the differences in side-leakage rate, *κ*_*s*_/*κ*, and the coupling strength, *g*/*κ* with fixed parameters of *γ*/*κ* = 0.1, *ω*_*c*_ = ${\omega }_{{{\rm{X}}}^{-}}$, and *ω* = *ω*_*c*_. The right plot represents the distribution of the values of AoF of the output state in terms of *κ*_*s*_/*κ* and *g*/*κ*. High efficiency and reliable performance (high AoF) of the interaction between a photon and QD-cavity system can be obtained when the strong coupling strength and small side-leakage rate, as described in table and plot. Also, the red-dotted boxes of plot and table show the distribution and values of AoF in the range of the large side-leakage rate, *κ*_*s*_/*κ*, and small coupling strength, *g*/*κ*. In the small range, the interactions of the QD-cavity system have the high efficiency and reliable performance (high AoF). But, because the range is small, the values of AoF rapidly decrease if the values of parameters (*κ*_*s*_/*κ* and *g*/*κ*) deviate a little from this range, as described in these plot and table.

In Fig. 9, the values of the AoF are listed for the difference in *κ*_*s*_/*κ* (*g*/*κ*), with fixed *g*/*κ* = 2.4 (*κ*_*s*_/*κ* = 2.0) in *γ*/*κ* = 0.1 and *ω* = *ω*_*c*_. The values of AoF approach 1 if the coupling strength is strong, *g* >> (*κ*, *γ*), and the side-leakage rate is small, *κ*_*s*_ << *κ*, as shown in Fig. 9. On the other hands, in the small range (0.1 ≤ *g*/*κ* ≤ 0.3 and 3.0 ≤ *κ*_*s*_/*κ* ≤ 4.5), we can acquire the high AoF (i.e., *g*/*κ* = 0.1 and *κ*_*s*_/*κ* = 4.5 → AoF ≈ 0.998). This means that the value of AoF can increase with small (weak) coupling strength and large side-leakage rate. However, when we experimentally implement the QD-cavity system for our scheme, it’s quite challengeable to maintain the values of parameters into the small range for the operation of the QD-cavity system. If the values of parameters deviate (less or over) from the small range, the value of AoF rapidly decreases, as described in Fig. 9, because of the tiny area of range. Rather, for the experimental realization, to improve the conditions (tendencies: strong coupling strength and small side-leakage) is more advantageous than to maintain the values of parameters fixed in the small range, in practice. Therefore, we can conclude that high efficiency and performance (according to the values of AoF) can be acquired by increase in the coupling strength, and also, with decrease in the side-leakage rate under the vacuum noise, *N*(*ω*), for operation of the QD-dipole and leaky modes, *S*(*ω*) (sideband leakage and absorption)^51,54,64–66^. Also, our optical Fredkin gate is utilizing the interactions of QD-cavity systems (QD1 and QD2) with high efficiency and performance when to increase the coupling strength, *g* >> (*κ*, *γ*), and decrease the side-leakage rate, *κ*_*s*_ << *κ*. If the conditions of QD-cavity systems for high values of AoF (approaching to 1) are maintained in our scheme, we can acquire the efficient controlled-swap operation from the interactions between a photon (control) and QDs (QD1 and QD2: targets) through the three cycles, as described in Figs. 4–6.

**Table 1:** In the table, the values of reflection, *R*_h_(*R*_0_), noise, *N*_h_(*N*_0_), and leakage, *S*_h_(*S*_0_), coefficients in hot (cold) cavity are listed for the differences in side-leakage rate, _k__s_/_κ_, with fixed the coupling strength, *g*/*κ* = 2.4, and also according to differences in *g*/*κ* with fixed *k*_s_/*κ* = 2.0, for fixed parameters of *γ*/*κ* = 0.1, and *ω* −*ω*_c_ = 0.

Reflection, Noise, and Leakage coefficients			Hot cavity			Cold cavity		
			*R*_h_(*ω*)	*N*_h_(*ω*)	*S*_h_(*ω*)	*R*_0_(*ω*)	*N*_0_(*ω*)	*S*_0_(*ω*)
*γ*/*κ* = 0.1 *ω* − *ω*_*c*_ = 0	*κ*_s_/*κ* ∵*g*/*κ* = 2.4	0.01	**0.991**	**0.131**	**−0.001**	**−0.980**	**0.000**	**−0.198**
		0.10	**0.991**	**0.131**	**−0.002**	**−0.818**	**0.000**	**−0.575**
		1.00	**0.991**	**0.131**	**−0.008**	**0.000**	**0.000**	**−1.000**
		2.00	**0.991**	**0.130**	**−0.012**	**0.333**	**0.000**	**−0.943**
	*g*/*κ* ∵*κ*_*s*_/*κ* = 2.0	0.01	**0.334**	**0.042**	**−0.941**	**0.333**	**0.000**	**−0.943**
		0.10	**0.412**	**0.378**	**−0.832**	**0.333**	**0.000**	**−0.943**
		1.00	**0.953**	**0.294**	**−0.065**	**0.333**	**0.000**	**−0.943**
		2.00	**0.987**	**0.155**	**−0.017**	**0.333**	**0.000**	**−0.943**

Moreover, for the high efficiency and performance of QD-cavity system in our scheme, the requirements of the reflection operator and interactions should be $\hat{{\rm{R}}}(\omega )$ in Eq. 4, and as Eq. 5. As shown In Table 1, we can calculate the values of the reflection, *R*_h_(*R*_0_), noise, *N*_h_(*N*_0_), and leakage, *S*_h_(*S*_0_), coefficients in hot (cold) cavity from Eqs. 2 and 20, according to the experimental parameters (*g*/*κ* and *κ*_*s*_/*κ*) with *γ*/*κ* = 0.1 and *ω* − *ω*_*c*_ = 0. Through the values in Table 1, we can compare with the reflection operators having high coupling strength and small leakage or not (i.e. *g*/*κ* = 2.4, *κ*_*s*_/*κ* = 0.01 or *g*/*κ* = 0.01, *κ*_*s*_/*κ* = 2.0), as follows:

$$[g/\kappa =2.4,\,{\kappa }_{s}/\kappa =0.01]:{\hat{{\rm{R}}}}_{1}\approx (1.121)|R\rangle \langle R|\otimes |\downarrow \rangle \langle \downarrow |+|L\rangle \langle L|\otimes |\uparrow \rangle \langle \uparrow |-(1.178)|R\rangle \langle R|\otimes |\uparrow \rangle \langle \uparrow |-|L\rangle \langle L|\otimes |\downarrow \rangle \langle \downarrow |,$$

24 $$[g/\kappa =0.01,\,{\kappa }_{s}/\kappa =2.0]:{\hat{{\rm{R}}}}_{2}\approx -(0.565)|R\rangle \langle R|\otimes |\downarrow \rangle \langle \downarrow |+|L\rangle \langle L|\otimes |\uparrow \rangle \langle \uparrow |-(0.610)|R\rangle \langle R|\otimes |\uparrow \rangle \langle \uparrow |-|L\rangle \langle L|\otimes |\downarrow \rangle \langle \downarrow |,$$

where the practical reflection operator, ${\hat{{\rm{R}}}}_{{\rm{P}}(\omega )}$, including noise, *N*(*ω*), and leakage, *S*(*ω*), coefficients is given by Eq. 21. These obviously mean that the practical reflection operator, ${\hat{{\rm{R}}}}_{1}$, is closer to the ideal reflection operator, $\hat{{\rm{R}}}$, in Eq. 4 when the coupling strength is strong, *g* >> (*κ*, *γ*), and the side-leakage rate is small, *κ*_*s*_ << *κ*, as shown in Fig. 9, Table 1, and Eq. 24, under the vacuum noise $\hat{N}$ in dipole operation and sideband leakage, absorption $\hat{S}$ in cavity.

Consequently, by our analysis, which is to quantify the efficiency and the reliable performance of the QD-cavity system via the reflection operator ${\hat{{\rm{R}}}}_{{\rm{P}}(\omega )}$ in Eq. 21, we demonstrate that the optical Fredkin (controlled-swap) gate using QD-cavity systems can be experimentally realized with feasibility.

## Conclusions

In this paper, we have proposed an optical scheme of Fredkin gate, which can realize controlled-swap gate for the interaction with a control qubit (photon) and two target qubits (electrons), using the QD-cavity systems. For the Fredkin gate, we also analyzed (quantified) the efficiency and performance (AoF: average of fidelity) of the QD-cavity system by calculating fidelity under the vacuum noise, *N*(*ω*), in the QD-dipole operation and leaky modes, *S*(*ω*) (sideband leakage and absorption)^51,54,64–66^, as described in Sec. 3. According to our analysis, when utilizing large coupling strength *g* >> (*κ*, *γ*) and low side-leakage rate *κ*_*s*_ << *κ*, we obtained high fidelity, F, of the output state from the QD-cavity system, due to the reduced (negligible) effect of the vacuum noise *N*(*ω*) and leaky modes *S*(*ω*).

Also, the methods have been proposed to implement Fredkin gate^23–26^. Milburn^23^ designed the optical Fredkin gate utilizing linearly optical devices under particular operating conditions (non-dissipative and error-free). But this scheme cannot be operated when to realize Fredkin gate in practice (dissipative and error). Afterward, many researchers have employed the cavity system (cavity QED) to compose Fredkin gate. In 2017, for the controlled-swap operation between a photon and atoms (hybrid system), Song *et al*.^24^ exploited (giant) Faraday rotation, which can be occurred from the difference in phase shift, according to the polarization of input state in hot or cold cavity, for the Fredkin gate. However, In practice, when to implement quantum information processing schemes using cavity system, various conditions, which can affect to diminish the performance, should be considered. Thus, they, in ref. ^24^, overlooked the affections of side-leakage rate (*κ*_*s*_), vacuum noise in QD-dipole operation, and leaky mode (sideband leakage and absorption) in the cavity mode. In ref. ^25^ a control qubit and target qubits correspond to a single superconducting flux and two resonator or nitrogen-vacancy (NV) centers in a hybrid Fredkin gate with quantum memories. Here, for the feasibility, our scheme utilized a single flying photon, as control qubit, because the photons can be used to best carriers for fast and reliable processing. Also, in ref. ^26^, the roles of NV centers are restricted to only the ancillary systems for the controlled-swap operation between three photons. Whereas, in our scheme, two electrons in QD-cavity systems (QD1 and QD2: target qubits) for the acquisition^43,45,46,55–57^ of the coherence of quantum state, when to perform the procedure of controlled operations. Thus, our optical scheme of Fredkin gate has the advantages (feasibility and efficiency), as the above mentions, compared with the previous works (the implementations of Fredkin gate)^23–26^.

In the point of the experimental implementation, to obtain such experimental condition for high fidelity, we reviewed previous related studies. In a micropillar cavity having diameter of 1.5 μm and quality factor as Q = 8800^68^, the coupling strength was achieved as *g*/(*κ* + *κ*_*s*_) ≈ 0.5, and also, could be increased to *g*/(*κ* + *κ*_*s*_)≈ 2.4 for Q = 40000^72^. Moreover, in ref. ^73^, by the technics (etching process or improving the sample growth), the authors could improve the experimental quality of optical cavity (In_0.6_Ga_0.4_As) as *g*/(*κ* + *κ*_*s*_) ≈ 2.4 and Q = 40000 by the decreasing rate of side-leakage. For small side-leakage rate, the method has been proposed to improve quality factor Q = 40000 (*κ* ≈ 6.2 μeV)^74^. Thus, QD-cavity systems in our scheme (optical Fredkin gate) can acquire the high efficiency and reliable performance (high fidelity), according to our analysis, in Sec. 3. Furthermore, the QD-cavity systems have provided the initialization, manipulation and measurement of the spin state for the interaction between an input photon (photonic spin) and a confined electron (electron spin) in QD, in^70^. Thus, for the preparation of electron spin-superposition state (arbitrary quantum state) in Fredkin gate, we can employ the methods as optical pumping and optical cooling^75^. And, the single spin of QD (the preparation of the excess electron spin state and operation on the spin) can be manipulated by using pulsed magnetic resonance techniques, nanosecond microwave pulses, or picosecond/femtosecond optical pulses^76–79^.

Consequently, based on our analysis, the QD-cavity system having the high efficiency and reliable performance can be operated in practice (under vacuum noise and sideband leakage). We have also demonstrated that controlled-swap operation of our Fredkin gate can be experimentally feasible by using the QD-cavity systems.
